# A knowledge-based scoring function for protein-RNA interactions derived from a statistical mechanics-based iterative method

**DOI:** 10.1093/nar/gku077

**Published:** 2014-01-28

**Authors:** Sheng-You Huang, Xiaoqin Zou

**Affiliations:** Department of Physics and Astronomy, Department of Biochemistry, Dalton Cardiovascular Research Center, and Informatics Institute, University of Missouri, Columbia, MO 65211, USA

## Abstract

Protein-RNA interactions play important roles in many biological processes. Given the high cost and technique difficulties in experimental methods, computationally predicting the binding complexes from individual protein and RNA structures is pressingly needed, in which a reliable scoring function is one of the critical components. Here, we have developed a knowledge-based scoring function, referred to as ITScore-PR, for protein-RNA binding mode prediction by using a statistical mechanics-based iterative method. The pairwise distance-dependent atomic interaction potentials of ITScore-PR were derived from experimentally determined protein–RNA complex structures. For validation, we have compared ITScore-PR with 10 other scoring methods on four diverse test sets. For bound docking, ITScore-PR achieved a success rate of up to 86% if the top prediction was considered and up to 94% if the top 10 predictions were considered, respectively. For truly unbound docking, the respective success rates of ITScore-PR were up to 24 and 46%. ITScore-PR can be used stand-alone or easily implemented in other docking programs for protein–RNA recognition.

## INTRODUCTION

Because of the importance of protein–RNA interactions on fundamental biological processes such as protein synthesis, DNA replication and repair, regulation of gene expression and defence against pathogens (1–8), determination of 3D protein–RNA complex structures would be valuable to understand the underlying recognition mechanisms at the atomic level (9–14). Despite the exponential growth in the experimental structures of individual proteins and RNAs in the Protein Data Bank (PDB) (15), the number of protein–RNA complex structures remains limited. As of 5 March 2013, there were only 1478 protein–RNA structures in the PDB. On the other hand, there are many more individual protein and RNA structures if we also count computationally modeled structures, such as structures constructed by homology modeling. Given the importance of protein–RNA recognition and the abundance of individual protein and RNA structures, computational methods for determination of the binding modes from individual protein and RNA structures, such as molecular docking (16–22) and template-based approaches (14), would have great potential for structural determination of protein–RNA complexes.

Although molecular docking for protein–protein recognition has been developed for more than one decade (23–41), the protein–RNA docking field is still in infancy and has received attentions only recently, partially motivated by the protein–RNA example in the Critical Assessment of PRedicted Interactions (CAPRI) experiments (42). This phenomenon may be attributed to several reasons. First, predicting the 3D structure of an RNA from its sequence is challenging. Unlike proteins for which there is a significant correlation between structure similarity and sequence homology, RNA molecules show much less conservation in primary sequences than in secondary and tertiary structures. Therefore, it is challenging to construct RNA structures from sequences through homology modeling, as shown in the exercise of Target 33 in the CAPRI experiment (42,43). Second, the numbers of experimentally determined RNA structures and protein–RNA bound structures are limited, which makes it more difficult to develop and assess protein–RNA docking algorithms than for protein–protein docking. Lastly, it is more challenging to predict conformational changes in RNA molecules than in proteins on binding because of the aforementioned less correlation between RNA sequences and structures.

To perform molecular docking, there are two equally important components, sampling and scoring. During the sampling process, all the possible binding modes of one molecule are sampled relative to the other molecule. Conformational changes may be considered during the sampling process. Following or during the sampling process, a scoring function is used to evaluate the energy associated with each generated binding pose and to rank the poses accordingly. The scoring function will be the focus of the present study. Although several attempts have been made recently toward the development of scoring functions for protein–RNA interactions (9–13,44–46), the scoring issue remains unsolved. No existing scoring function has been extensively tested owing to the lack of a large diverse test set of protein–RNA structures in the past (47–49). Several factors should be considered for the development of a scoring function for protein–RNA interactions. First, owing to the huge number of possible binding modes that are sampled, particularly for global sampling, a fast and efficient scoring function is needed to complete the energy evaluation process within a practical period with reasonable accuracy. A second challenge in scoring function development is protein/RNA flexibility and structural distortion. Specifically, conformational changes often occur on protein–RNA binding. Also, given the limited number of individual protein and RNA structures, theoretical structures may also be used for docking. Therefore, a scoring function should be able to handle a certain degree of molecular distortion arising from binding-induced conformational changes and modeling-related inaccuracies. Lastly, a large and diverse structural data set is needed for scoring function validation.

Recently, we have developed a statistical mechanics-based method for scoring function construction by extracting atomic, distance-dependent interaction potentials from experimentally determined structures (50). The method circumvents the long-standing ‘reference state’ issue (51–55) in knowledge-based scoring functions by using a physics-based iterative algorithm, and has proved to be efficient on scoring/ranking protein–protein binding decoys (43,56) in the CAPRI experiments (42). In this study, we have developed a scoring function based on the atomic distance-dependent potentials derived from known protein–RNA structures, referred to as ITScore-PR, for predicting protein–RNA complex structures from individual unbound protein/RNA structures. The scoring function has been tested for its ability to identify near-native binding modes by using four test sets that were prepared by different docking methods and benchmarks. The scoring function and the decoy set generated for our validation study are expected to be beneficial to the development of computational algorithms for protein–RNA docking.

## MATERIALS AND METHODS

### Training set for deriving potentials

To construct a diverse training set of protein–RNA complex structures, we searched the PDB for all the radiographic crystal structures that have a resolution better than 3.5 Å and contain at least one protein and one RNA chain but no DNA chains. As of 7 December 2012, the query yielded 962 entries, which were further manually examined. Only the appropriate protein–RNA complexes that met the following criteria were considered. First, both the protein and RNA chains should belong to the same biological unit according to the description in the PDB file. Second, the individual protein and RNA should be large enough to have a stable structure, but not too large for docking calculations. In this study, the number of the residues in the protein was set to be >20 and <1000, and the number of the residues in the RNA was between 10 and 200. Third, to ensure that the binding interface forms a whole patch rather than multiple separate patches for the sake of simplicity, only the PDB entries that contain a moderate number of chains in the protein oligomers or multiple RNA segments were considered. Specifically, we empirically allowed for no more than six chains in the protein or RNA. Finally, the complexes with only backbone atoms in the protein or in the RNA were excluded. The complexes that met the above criteria were then clustered according to their sequence similarities to remove the redundancy. Namely, two protein–RNA complexes were grouped into the same cluster if they met the following two criteria: First, any protein chain of the first complex has a sequence identity >30% with a protein chain from the second complex. Second, any RNA chain of the first complex has a sequence identity >70% with an RNA chain from the second complex. The higher sequence identity cutoff for RNA during clustering was because RNA molecules have a much lower sequence-structure homology than proteins (57). A total of 175 clusters of protein–RNA complexes were obtained. The crystal structure with the best resolution in each cluster was selected as a representative.

These 175 protein–RNA complex structures were found to have some overlap with the protein–RNA docking benchmark 1.0 that we developed recently (49). Considering that the benchmark will be used as a test set to validate our scoring function, we eliminated the overlap by removing the complexes in the training set that have >30% sequence identity with the protein chain and >70% sequence identity with the RNA chain of a complex in the benchmark. The resulting training set contained 110 diverse protein–RNA complex structures, which were used to derive our scoring function. The PDB entries of these 110 complexes are listed in Supplementary Table S1.

### Statistical mechanics-based method to derive the interaction potentials

We used a statistical mechanics method to derive the interaction potentials between the protein and the RNA from a training set of diverse protein–RNA complex structures. Our method circumvents the challenging ‘reference state’ problem in knowledge-based scoring functions. The basic idea behind the method is to improve the interatomic pair potentials step by step though iterations by comparing the predicted pair distribution functions of the protein–RNA complexes and the experimentally observed pair distribution functions of the (native) crystal structures in the training set. The method can be represented mathematically by the following iterative formula:
(1)
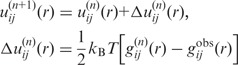

where *n* stands for the iterative step, *i* and *j* represent the types of a pair of atoms in the protein and the RNA, respectively. 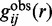 stands for the pair distribution function for atom pair *ij* calculated from the experimentally observed protein–RNA complex structures in the training set:
(2)
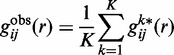

where *K* is the total number of the protein–RNA complexes in the training data set and 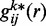 is the pair distribution function of the *k*-th native complex structure (50,56,58,59). The 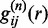 is the pair distribution function calculated from the ensemble of the binding modes according to the binding score-dependent Boltzmann probabilities 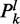 (50) that are predicted with the trial potentials 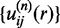 at the *n*-th step.
(3)
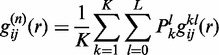

where 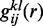 is the pair distribution function for atom pair *ij* observed in the *l*-th binding state of the *k*-th protein–RNA complex (50,56). 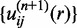 are the improved potentials from 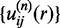 after the correction and are used in the next iterative step. Without loss of generality, 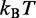 was set to unit 1 in the iterations.

Theoretically, 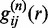 can be calculated from an ensemble of binding modes generated by Monte Carlo (MC) simulations with the potentials 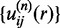 at each iterative step. However, given the large number of complexes and atoms therein, running MC simulations to sample a complete set of binding modes at each cycle would be computationally impractical. Therefore, an ensemble of globally sampled binding orientations is pre-generated for the iteration process. Specifically, to avoid any bias, the third-party docking software ZDOCK 2.1 (30) was used to generate a set of possible binding modes. ZDOCK 2.1 uses a basic shape complementarity scoring function. All the default parameters of ZDOCK 2.1 were used during docking. A total of 2000 binding modes were generated by default.

Next, a simplex optimization (60) was performed to remove atomic clashes in the binding modes sampled by ZDOCK 2.1 by using a van der Waals (VDW) scoring function. The top 1000 binding modes based on the VDW scores plus one native structure were used as the structural ensemble for the whole iteration procedure.

Then, for a given set of initial potentials 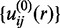,
(4)
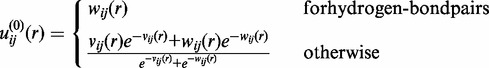

where 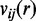 is the 6-12 VDW potential and 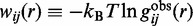 is the potential of mean force (56). The first iterative step will lead to an improved set of pair potentials 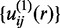 via the Equation (1), followed by 
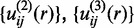
 and so on in the following steps. The iteration continues until all the native binding structures were discriminated from the decoys by the current potentials.

The final set of pair potentials were treated with the following smoothing algorithm to account for statistical fluctuations in the experimentally determined structures in the training set: The potential at the *i*-th bin was set to the weighted average of 1:2:4:2:1 of the potentials from bins 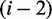 to 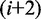.

**Figure 1.**
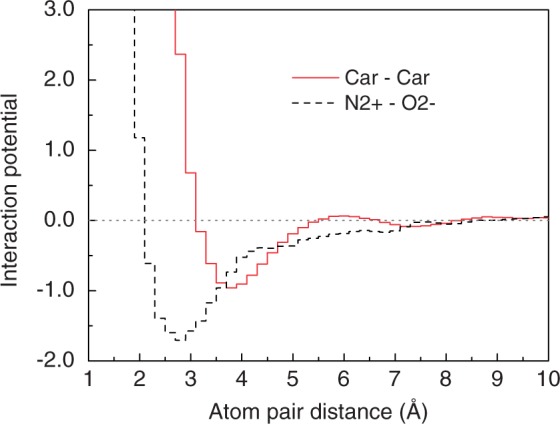
Two example pair potentials for ITScore-PR.

In this study, only heavy atoms were considered and the effects of hydrogens were implicitly incorporated in the potentials. The categorization for the protein atom types is the same as in our previous study for ITScorePP (56), giving 20 atom types. The categorization for the RNA atom types was based on our method for ITScore (58), yielding 12 atom types (Table 1). The VDW radii and well depths for the calculation of the initial potentials were taken from (56). The radius of the reference sphere used for pair distribution function calculations was set as 12 Å. The bin size 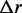 for the distance was set at 0.2 Å. The cutoff distance 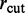 of the potentials for binding score calculations was set to 10 Å, as the potentials approach zero at this distance (Figure 1). The maximum penalty for the potentials at short distances was set 100 kcal/mol unless otherwise specified.

**Table 1.** List of 20 protein atom types and 12 RNA atom types used in ITScore-PR, in which ‘*’ stands for any residue name of proteins

Numbers	Symbol	Atom name
Protein		
1	C2+	ARG_CZ
2	C2-	ASP_CG, GLU_CD
3	C2M	*_C
4	C2S	ASN_CG, GLN_CD
5	Car	HIS_CD2, HIS_CE1, HIS_CG, PHE_CD1, PHE_CD2, PHE_CE1, PHE_CE2, PHE_CG, PHE_CZ, TRP_CD1, TRP_CD2, TRP_CE2, TRP_CE3, TRP_CG, TRP_CH2, TRP_CZ2, TRP_CZ3, TYR_CD1, TYR_CD2, TYR_CE1, TYR_CE2, TYR_CG, TYR_CZ
6	C3C	ALA_CB, ARG_CB, ARG_CG, ASN_CB, ASP_CB, GLN_CB, GLN_CG, GLU_CB, GLU_CG, HIS_CB, ILE_CB, ILE_CD1, ILE_CG1, ILE_CG2, LEU_CB, LEU_CD1, LEU_CD2, LEU_CG, LYS_CB, LYS_CD, LYS_CG, MET_CB, PHE_CB, PRO_CB, PRO_CG, THR_CG2, TRP_CB, TYR_CB, VAL_CB, VAL_CG1, VAL_CG2
7	C3A	*_CA
8	C3X	ARG_CD, CYS_CB, LYS_CE, MET_CE, MET_CG, PRO_CD, SER_CB, THR_CB
9	N2N	ALA_N, ARG_N, ASN_N, ASP_N, CYS_N, GLN_N, GLU_N, GLY_N, HIS_N, ILE_N, LEU_N, LYS_N, MET_N, PHE_N, PRO_N, SER_N, THR_N, TRP_N, TYR_N, VAL_N
10	N2+	ARG_NH1, ARG_NH2
11	N2X	ASN_ND2, GLN_NE2
12	Nar	HIS_ND1, HIS_NE2, TRP_NE1
13	N21	ARG_NE
14	N3+	LYS_NZ
15	O2M	*_O
16	O2S	ASN_OD1, GLN_OE1
17	O3H	SER_OG, THR_OG1, TYR_OH
18	O2-	ASP_OD1, ASP_OD2, GLU_OE1, GLU_OE2
19	S31	CYS_SG
20	S30	MET_SD
RNA		
1	C2X	C_C2, G_C6, U_C2, U_C4
2	Car	C_C4, C_C5, C_C6, G_C2, U_C5, U_C6, A_C2, A_C4, A_C5, A_C6, A_C8, G_C4, G_C5, G_C8
3	C3X	A_C1’, A_C2’, A_C3’, A_C4’, A_C5’, C_C1’, C_C2’, C_C3’, C_C4’, C_C5’, G_C1’, G_C2’, G_C3’, G_C4’, G_C5’, U_C1’, U_C2’, U_C3’, U_C4’, U_C5’
4	N2N	C_N1, G_N1, U_N1, U_N3
5	N2X	A_N6, C_N4, G_N2
6	Nar	C_N3, G_N3, A_N1, A_N3, A_N7, G_N7
7	N21	A_N9, G_N9
8	O2	C_O2, G_O6, U_O2, U_O4
9	O31	A_O2’, C_O2’, G_O2’, U_O2’
10	O32	A_O3’, A_O4’, A_O5’, C_O3’, C_O4’, C_O5’, G_O3’, G_O4’, G_O5’, U_O3’, U_O4’, U_O5’
11	O2-	A_OP1, A_OP2, C_OP1, C_OP2, G_OP1, G_OP2, U_OP1, U_OP2
12	P	A_P, C_P, G_P, U_P

### Test sets for validating ITScore-PR

In this study, we have used four test sets that were constructed by different groups using different algorithms to test the performance of ITScore-PR on identification of near native binding modes for both bound and unbound docking.

#### The ROSETTA docking decoys prepared by the Varani Group

This test set consists of five protein–RNA complexes (PDB codes: 1CVJ, 1EC6, 1FXL, 1JID and 1URN). For each complex, 2000 binding decoys were generated by Chen et al. in the Varani Group (9) using the protein–protein docking module (61) of ROSETTA to test their knowledge-based potentials (9,10), in which the RNA molecule was treated as a rigid body and the protein backbone was also fixed. The rigid RNA molecule was first perturbed around the binding site on the protein, followed by a protein side chain repacking and optimization for each relative translation and orientation of the two partners. This set is regarded as a semi-unbound (or semi-bound) test set because the decoys generated from this protocol include both rigid (i.e. the RNA and protein backbones) and flexible (i.e. the side chains) components. The final decoys cover a wide range of root mean square deviation (RMSD) values from 0.2 Å to ∼30 Å relative to the corresponding native complexes.

#### Protein–RNA docking benchmark constructed by Huang and Zou

The second test set for this study is the protein–RNA docking benchmark 1.0 (49) that we recently developed (http://zoulab.dalton.missouri.edu/RNAbenchmark/). Briefly, the benchmark contains 72 diverse targets of protein–RNA complex structures. Each target includes both the bound structures and unbound structures of the protein and the RNA. The unbound structure is defined as a structure in free form or being a binding partner in a different complex. In addition, following the sequence alignment, a residue mapping between the bound and unbound structures was obtained for both the protein and the RNA, respectively. Based on the residue mapping, a second set of mapped bound and unbound structures was created by removing the mismatched residues in the alignments from the original structure files. The mapped bound and unbound structures are important for binding mode quality assessment. The benchmark also contains other useful information, such as the interface RMSD between the bound complex and the unbound complex after optimal superimposition and the percentage of native contacts of the unbound complex compared with the bound complex. The information provides the benchmark users a clear picture about the degree of conformation changes on RNA binding. Detailed description about the benchmark can be found in our protein–RNA benchmark paper (49). This benchmark was used for bound and unbound docking tests in the present study.

#### Protein–RNA docking benchmark constructed by Perez-Cano et al

The third test set is the protein–RNA docking benchmark constructed by Perez-Cano et al. in the Fernandez-Recio Group using a different set of rules (http://life.bsc.es/pid/protein-rna-benchmark/) (48). The authors strictly considered only the structures in free form as unbound structures, except for pseudo-unbound RNA structures that are bound to a nonhomologous protein because of the limited number of free RNA structures in the PDB. For the cases with no unbound structures available, the authors used modeled structures that were built based on the homologous templates with a sequence identity of 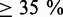 for proteins and 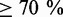 for RNA. By including the modeled structures, the benchmark contains a large set, with 106 cases. Among them, 71 cases have both bound and unbound experimentally determined structures, and the remaining 35 cases have at least one of the unbound structures modeled by homology. To remove the overlap between this benchmark and our training set, we excluded the homologous test cases with sequence identity >30% for the proteins and >70% for the RNA molecules with respect to the protein–RNA complexes in our training set. This process reduced the number of the test cases to 78. We further removed five cases of biological assembly and one case in which the RNA residues are swapped between the target and template structures. A final set of 72 protein–RNA test cases were obtained, as described in Section ‘Test on the protein–RNA docking benchmark prepared by Perez-Cano et al.’ and Supplementary Table S9. The test set served as an assessment of ITScore-PR with the structures prepared by different rules and protocols.

#### The RPDOCK docking decoys prepared by Huang et al

This test set consists of two sets of decoys that were generated by Huang et al. in the Xiao Group using their RPDOCK program (62). The first decoy set was constructed based on the protein–RNA docking benchmark by Perez-Cano *et al.* (48), and the second set was based on the protein–RNA docking benchmark by Huang and Zou (49) (Supplementary Table S2). Specifically, first, the authors used the RPDOCK program to generate the binding decoys based on the unbound/modeled structures in the benchmarks. Only those test cases with at least one near-native poses in the top 1000 decoys were kept, where the near-native pose was defined as the pose with an RMSD of the RNA molecule <10 Å compared with the corresponding native complex after the superimposition of the proteins. This protocol resulted in 43 test cases for the decoy set based on the benchmark by Perez-Cano *et al.* (48), and 50 test cases for the decoy set based on the benchmark by Huang and Zou (49). These two decoy sets were also used in the present study to assess ITScore-PR on the structures generated from different docking protocols.

### Criteria for the assessment of the prediction quality

The assessment of the prediction quality was based on three parameters that were used in CAPRI: 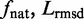 and 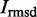 (42,63–65). 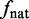 stands for the percentage of native residue–residue contacts in the predicted binding mode relative to the total residue contacts in the crystal structure. Here, a contact is defined as a pair of residues whose nearest atom pair is within 5.0 Å. 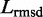 is the ligand (RNA) RMSD between the predicted binding mode and the native structure after the corresponding receptors (proteins) are optimally superimposed. 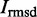 is the RMSD of the interface region between the predicted binding complex and the native structure after optimal superimposition. Here, the interface is defined as the contacting residues in the native protein–RNA structure that are within 10 Å and belong to different binding partners. In the evaluation, all the superimpositions and RMSD calculations were based on Cα atoms for proteins and C4^′^ atoms for RNA molecules, unless otherwise specified (49).

According to the above three parameters, the prediction quality is classified into four categories: high accuracy, medium accuracy, acceptable accuracy and incorrect prediction. Details are explained in our previous study (56) or the CAPRI analysis papers (63,64). In the present work, we defined that the binding mode of a protein–RNA complex is successfully predicted if the best-scored (i.e. the top) RNA orientation has at least acceptable accuracy. This criterion is the default success criterion unless otherwise specified.

## RESULTS AND DISCUSSION

### Extracted effective pair potentials

Using the 1001 binding modes (1000 decoys plus one native structure) for each of the 110 protein–RNA complexes in the training set, we have extracted the effective pairwise potentials for protein–RNA interactions with our statistical mechanics-based iterative method. The 20 protein atom types and 12 RNA atom types, which are listed in Table 1, may result in 240 different pairs. The number of occurrences within the reference sphere (see ‘Materials and Methods’ section) depends on the pair of protein and RNA atom types, as shown in Supplementary Table S3. For example, the number of occurrences was 163 501 for C3C-C3X, and 33 528 for Car-Car. To guarantee sufficient statistics, only the atom type pairs with >1000 occurrences were retained, resulting in 206 pairs of effective interaction potentials out of 240 possible pairs. The iteration process was efficient and normally converged within 10 cycles, as shown in Supplementary Figure S1. Supplementary Figure S1 shows two example pairwise potentials. Several notable features can be found in the potentials and are consistent with experimental findings. For the atom pair Car–Car, the potential curve exhibits an energy minimum at ∼3.6 Å, representing the hydrophobic interaction between the atom pair. For the atom pair N+–O−, the energy minimum occurs at ∼2.8 Å, which is consistent with the corresponding hydrogen-bonding interaction. The more favorable (negative) potential for the N+–O− pair is due to the attractive electrostatic interaction between these two oppositely charged atom types in addition to the hydrogen-bonding interactions.

### Test on the ROSETTA docking decoys

**Figure 2.**
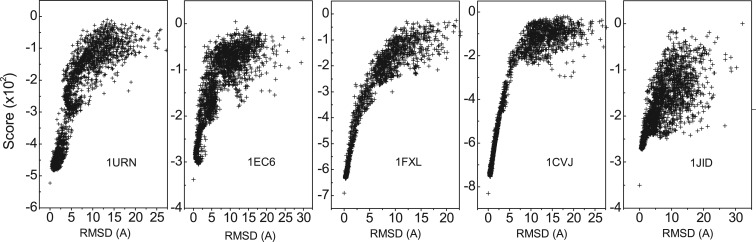
The score-RMSD plots of ITSocre-PR for the ROSETTA docking decoys (five complexes) generated by the Varani group (9).

The derived ITScore-PR was evaluated on each of the ROSETTA decoys generated by Chen *et al.* (9) in the Varani Group. Figure 2 shows the score-RMSD plots for the five test cases. It can be seen from the figure that all of the native structures were identified, which were associated with the lowest ITScore-PR scores with respect to the decoys. To quantify the ability of ITScore-PR to identify near-native protein–RNA structures, we calculated the score-RMSD correlation coefficients for the decoys with RMSD <5 Å, <10 Å and <20 Å, respectively. The results are listed in Supplementary Table S4. For references, we calculated the corresponding correlation coefficients for dRNA, a knowledge-based scoring function from the Zhou Group (14). We also took the correlation results of four other knowledge-based scoring functions from the study by Tuszynska and Bujnicki (45): DARS-RNP and QUASI-RNP by Tuszynska and Bujnicki (45), the potentials derived by the Varani Group (10) and the potentials derived by the Fernandez-Recio Group (11). It can be seen from the table that there exist strong score-RMSD correlations for ITScore-PR, dRNA, DARS-RNP and QUASI-RNP, with an average correlation coefficient of 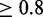 for all the RMSD ranges. Compared with other scoring functions, ITScore-PR performed relatively better on the test case 1URN, with the correlations of 0.84, 0.89 and 0.84, followed by DARS-RNP with the correlations of 0.77, 0.83 and 0.81, when the decoys of RMSD <5 Å, <10 Å and <20 Å were considered, respectively. Overall, ITScore-PR performed slightly better (correlations of 0.86, 0.89 and 0.84 for RMSD <5 Å, <10 Å and <20 Å, respectively), followed by dRNA (0.81, 0.88 and 0.86), DARS-RNP (0.81, 0.88 and 0.85) and QUASI-RNP (0.80, 0.87 and 0.84). The respective correlations were 0.53, 0.47 and 0.39 for the Varani potential, and 0.23, 0.35 and 0.38 for the Fernandez potential. In summary, ITScore-PR is able to identify the near-native structures by achieving strong score-RMSD correlations for all the five test cases of the ROSETTA docking decoys.

### Test on the protein–RNA docking benchmark prepared by Huang and Zou

ITScore-PR was also tested for its ability to distinguish near-native structures from binding decoys with a wide range of RMSDs using the protein–RNA benchmark of 72 complexes developed by our group (49), for both bound docking and unbound docking. Here, bound docking refers to rigid redocking, namely, reproducing the co-crystalized structure by using individual separated bound conformations. The advantage for the use of bound orientations is the absence of molecular flexibility so that bound docking is a direct and essential assessment of a scoring function. Unbound docking refers to the use of an apo structure or a conformation taken from a different protein–RNA complex for docking, following the commonly used definition in the protein–protein docking field (66,67).

#### Validation protocol

The validation process is described as follows. First, for each protein–RNA complex in the test set, 2000 binding orientations of the RNA molecule were generated globally relative to the protein for the bound conformations and unbound conformations, respectively, using the third-party docking software ZDOCK 2.1 (30). ZDOCK 2.1 uses a simple shape complementarity scoring function and thus would introduce the least bias toward sampled conformations. Here, we use bound cases as an example. Specifically, using the bound conformation of each complex in the test set, the possible orientations were sampled globally and rigidly via ZDOCK 2.1, and 2000 orientations were kept. The same procedure was repeated for unbound cases. Considering that ZDOCK 2.1 might be unable to generate near-native binding orientations in the 2000 poses, we included the native bound structures for bound docking and the superimposed unbound structures for unbound docking for the scoring function assessment. Namely, there are 2001 RNA bound orientations (i.e. 2000 bound decoys plus one native pose) and 2001 RNA unbound orientations (i.e. 2000 unbound decoys plus one superimposed unbound structure). These 2001 RNA bound (or unbound) decoys were then evaluated with ITScore-PR, during which the simplex algorithm (60) was used to optimize the ITScore-PR scores. The refined decoys by ITScore-PR covered the whole protein surface and included a certain number of near-native hits in all the bound cases and in most of the unbound cases (see Supplementary Table S5 and Figures S2 and S3), which may serve as a useful decoy set for scoring function assessment on protein–RNA docking. Lastly, for each complex, all the orientations were ranked according to their ITScore-PR scores and clustered based on their RMSD distances. If two orientations have an RMSD of <5 Å for the heavy atoms in the RNA molecule, only the orientation with a lower ITScore-PR score was kept. For consistency, the same clustering strategy was also applied to the ranked orientations during the calculations of the success rates for other scoring functions being tested in this study.

#### The bound cases

**Figure 3.**
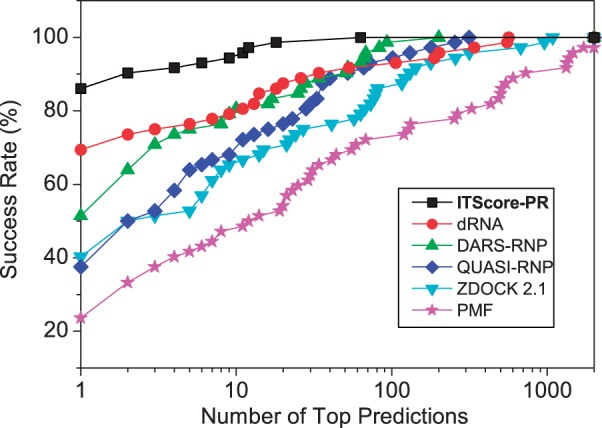
The success rates of ITScore-PR and five other scoring functions (dRNA, DARS-RNP, QUASI-RNP, ZDOCK 2.1 and PMF) as a function of the number of top ranked orientations for the bound test cases of the 72 complexes in the protein–RNA docking benchmark by Huang and Zou (49).

Figure 3 shows the success rates of bound docking as a function of the number of the top RNA orientations ranked by ITScore-PR (referred to as top predictions) based on the CAPRI criteria (63). For validation purpose, the results of five other scoring functions, dRNA (14), DARS-RNP, QUASI-RNP (45), ZDOCK 2.1 (30) and pure PMF, are also listed in the figure. ZDOCK 2.1, which was originally developed for protein–protein docking, is approximated as a shape complementary-based scoring function for protein–RNA docking. The pure PMF refers to the traditional approximation of the reference state by randomly mixing all the atoms in the training set. It can be seen from the figure that ITScore-PR shows significantly better performance in binding mode prediction and yielded a success rate of 86.1% if only the top ranked orientation was considered, compared with 69.4% for dRNA, 51.4% for DARS-RNP, 37.5% for QUASI-RNP, 40.3% for ZDOCK 2.1 and 23.6% for PMF (Supplementary Figure S4). If the top 10 ranked orientations were considered, ITScore-PR achieved a success rate of 94.4%, compared with 79.2% for both dRNA and DARS-RNP, 68.1% for QUASI-RNP, 65.3% for ZDOCK 2.1 and 46.2% for PMF (Supplementary Figure S4).

To analyze the accuracy of ITScore-PR in more details, we also examined the accuracy qualities and the rankings of the first successful predictions for the benchmark of 72 bound cases. As shown in Supplementary Table S6, most of the successful binding modes predicted by ITScore-PR were high-accuracy structures according to the CAPRI criteria and were ranked as No. 1, whereas the other scoring functions yielded relatively less accurate modes (i.e. more models with medium or acceptable accuracy) and lower rankings for the best successful predictions. PMF was even unable to predict any successful binding modes for two bound cases (Supplementary Table S6).

#### The unbound cases that include homologous unbound structures

Next, ITScore-PR was assessed using the ‘unbound’ cases of these 72 complexes in which at least one partner of each complex has a nonnative structure (i.e. either an apo structure or a structure taken from a different protein–RNA complex) (49), following the definition used in the protein–protein docking field (66,67). Some of these ‘unbound’ structures are homologous to the corresponding bound structures. Unbound docking is considered to be a realistic assessment for a scoring function because of the involvement of conformational changes and uncertainties in structural modeling. Current docking algorithms are still not sophisticated enough to accurately handle molecular flexibility during docking calculations. Therefore, scoring functions should be robust enough to be able to implicitly account for partial induced conformational changes without much sacrifice of its accuracy to make reasonable binding mode predictions. To this end, softer pairwise potentials were introduced for ITScore-PR for unbound docking. Namely, the potential penalty at short distances was reduced to 10 kcal/mol, allowing for limited tolerance on severe atomic clashes between the protein and the RNA during unbound docking.

**Figure 4.**
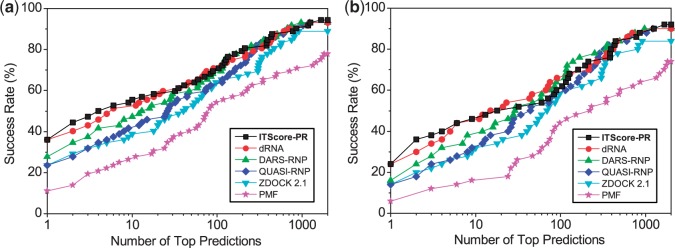
The success rates of ITScore-PR and five other scoring functions (dRNA, DARS-RNP, QUASI-RNP, ZDOCK 2.1 and PMF) as a function of the number of top ranked orientations for (**a**) all the unbound cases in which homologous unbound structures are included (72 complexes), and (**b**) the ‘truly’ unbound test cases of the 50 complexes from the protein–RNA docking benchmark by Huang and Zou (49). The details are explained in the text.

Figure 4a shows a comparison of the success rates of ITScore-PR and five other scoring functions (dRNA, DARS-RNP, QUASI-RNP, ZDOCK 2.1 and PMF) for the unbound cases of the 72 protein–RNA targets. When the top ranked orientation was considered, ITScore-PR and dRNA yielded the same success rate with 36.1%, compared with 27.8% for DARS-RNP, 23.6% for both QUASI-RNP and ZDOCK 2.1 and 11.1% for PMF (Supplementary Figure S5a). When the top 10 predictions were considered, the success rate of ITScore-PR increased to 55.6%, compared with 51.4% for dRNA, 47.2% for DARS-RNP, 41.7% for QUASI-RNP, 38.9% for ZDOCK 2.1 and 26.4% for PMF (Supplementary Figure S5a).

**Figure 5.**
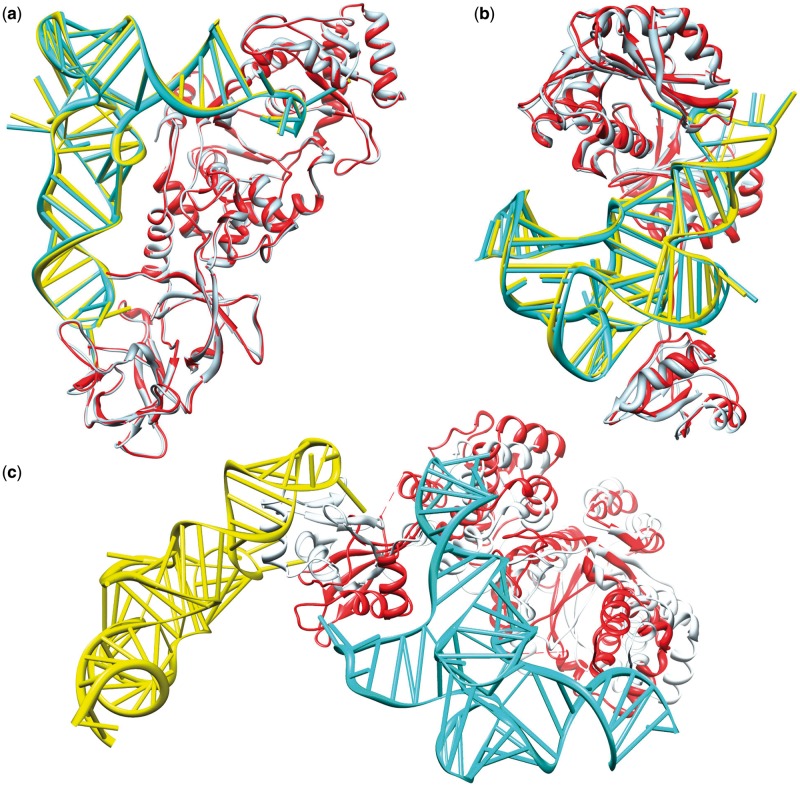
The comparison between the predicted complex (protein: light blue, RNA: yellow) and experimentally determined crystal structure (protein: red, RNA: cyan) of three selected unbound test cases: (**a**) 1QTQ (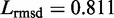 Å, 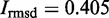 Å and 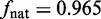), (**b**) 2ZZM (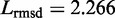 Å, 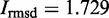 Å and 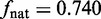) and (**c**) 1H3E (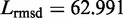 Å, 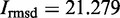 Å and 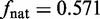).

Figure 5 shows the predicted binding modes for three example unbound docking cases; each case belongs to a different difficulty category in the protein–RNA docking benchmark (49). It can be seen from the figure that ITScore-PR gave high-accuracy predictions for 1QTQ (‘easy target’), with 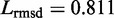 Å, 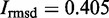 Å and 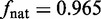. ‘Medium target’ 2ZZM was predicted at medium accuracy, with 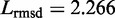 Å, 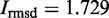 Å and 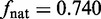. Even for the ‘difficult target’ 1H3E, ITScore-PR was still able to achieve a medium-accuracy prediction (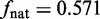) and recognized the flexible C-terminal domain of the tyrosyl-tRNA synthetase. Remarkably, this domain flips its orientation with an RMSD as large as 25.87 Å (see Figure 5c) and plays a crucial role in the recognition of tRNA^tyr^ (68).

We also analyzed the accuracy qualities and rankings of the first successful predictions for the 72 unbound cases of the benchmark (Supplementary Table S7). Similar to the bound cases, ITScore-PR yielded relatively higher accuracy predictions and lower rankings for the first predicted successful modes than the other scoring methods like ZDOCK2.1 and PMF, even though the improvement is relatively less than bound docking because of the effect of molecular flexibility.

#### The unbound cases in which homologous unbound structures are excluded

To further test ITScore-PR, we created a subset of ‘truly’ unbound cases from the 72 protein–RNA complexes in our protein–RNA docking benchmark by removing those homologous unbound cases. Here, ‘truly’ unbound cases are defined as the cases in which at least one of the binding partners is a ‘truly’ unbound structure, either in the free form or bound to an RNA (or a protein) that has a sequence identity of <70% (or <30%) compared with the RNA (or the protein) in the corresponding native complex. We ended with 50 truly unbound test cases out of the original 72 cases (Supplementary Table S8).

Figure 4b shows the success rates of ITScore-PR, dRNA, DARS-RNP, QUASI-RNP, ZDOCK 2.1 and PMF for these 50 truly unbound cases. A similar trend of performance can be found as compared with Figure 4a, despite the decrease of the success rates for all the scoring functions. If the top ranked orientation was considered, ITScore-PR tied with dRNA, both having a success rate of 24%, compared with 16% for DARS-RNP, 14% for QUASI-RNP, 14% for ZDOCK 2.1 and 6% for PMF (Supplementary Figure S5b). If the top 10 predictions were considered, the success rate of ITScore-PR increased to 46%, compared with 44% for dRNA, 38% for DARS-RNP, 32% for both QUASI-RNP and ZDOCK 2.1 and 16% for PMF (Supplementary Figure S5b).

The success rates are lower for the subset of truly unbound cases than for the original set of 72 complexes (Figure 4) are expected because homologous complexes tend to adopt similar conformations. In other words, the unbound structures that are taken from the homologous complexes would be closer to the native bound conformations and therefore make the corresponding unbound test cases easier for prediction.

The similar performance of ITScore-PR and dRNA on the unbound test cases as shown in Figure 4 may be partially attributed to the facts that both scoring functions are knowledge-based and have been optimized by addressing the reference state issue. ITScore-PR circumvents the reference state problem via an iterative method and dRNA improves the reference state approximation by introducing a volume correction. In addition, the decoys prepared by using ITScore-PR are optimized and contain no atomic clashes, which excludes close atomic contacts and may help the performance of the reference state-based scoring functions including dRNA. Regarding the differences, for bound docking, ITScore-PR would achieve a significantly higher success rate than dRNA. For general unbound cases, ITScore-PR performed slightly better than dRNA when only a few top conformations were considered (also see the following subsections). It is noted that all these unbound studies treated the molecular flexibility implicitly. It would be interesting for future studies to make comparative assessment of the performances of these scoring functions when they are combined with good conformational sampling algorithms that are able to handle conformational changes properly.

### Test on the protein–RNA docking benchmark prepared by Perez-Cano et al

**Figure 6.**
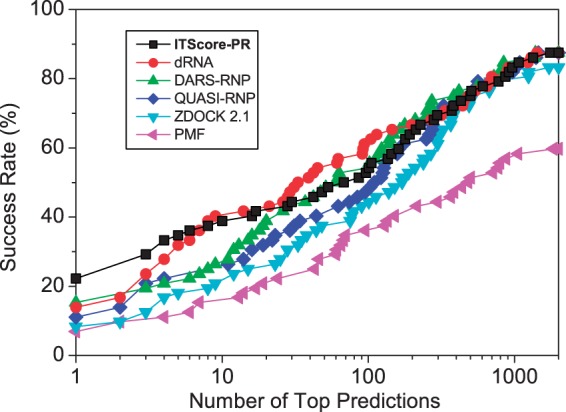
The success rates of ITScore-PR and five other scoring functions (dRNA, DARS-RNP, QUASI-RNP, ZDOCK 2.1 and PMF) as a function of the number of top ranked orientations for the unbound test cases of the 72 complexes from the protein–RNA docking benchmark by Perez-Cano *et al.* (48).

ITScore-PR was further tested on the 72 complexes from the protein–RNA docking benchmark constructed by Perez-Cano *et al.* (48), which contain no complexes that are homologous to the complexes in our training set. We used the same decoy generation method and validation protocol as described in the previous section. Only the unbound/model cases were studied for this set. Figure 6 shows the success rates of ITScore-PR as a function of the number of top predictions. The corresponding rankings and accuracy qualities of the first successful predictions were listed in Supplementary Table S9. For comparison, the success rates of five other scoring methods, dRNA, DARS-RNP, QUASI-RNP, ZDOCK 2.1 and PMF, were also plotted in Figure 6. It can be seen from the figure that if the top few predictions were considered, which is often the case in realistic applications, ITScore-PR yielded higher success rates than the other five scoring functions. If the top prediction was considered, ITScore-PR performs significantly better with a success rate of 22.2%, compared with 13.9% for dRNA, 15.3% for DARS-RNP, 11.1% for QUASI-RNP, 8.3% for ZDOCK 2.1 and 6.9% for PMF (Supplementary Figure S6). If the top 10 predictions were considered, both ITScore-PR and dRNA obtain a good success rate of ∼40% that is significantly higher than DARS-RNP (26.4%), QUASI-RNP (22.2%), ZDOCK 2.1 (20.8%) and PMF (15.3%) (see Supplementary Figure S6).

### Test on the RPDOCK docking decoys

Xiao and coworkers constructed two decoy sets using their RPDOCK docking protocol (62). The first set was based on the 43 unbound test cases in the protein–RNA benchmark by Perez-Cano *et al.* (48), and the second set was based on the 50 unbound cases in the protein–RNA benchmark by Huang and Zou (49). To make the results comparable, we adopted the same criterion used in (62) to measure the success of predictions for this test. Specifically, a decoy is defined as a successful prediction if the RMSD of the RNA molecule is <10 Å from the native structure after the superimposition of the proteins. Neither did we cluster the scored decoys by ITScore-PR so that our results can be directly compared with the results in (62).

**Figure 7.**
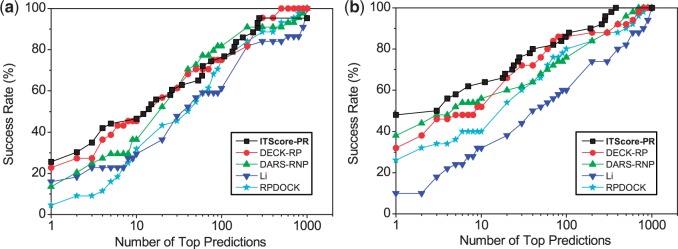
The success rates of ITScore-PR and four other scoring functions (DECK-RP, DARS-RNP, the Li potential and RPDOCK) as a function of the number of top ranked orientations for the RPDOCK docking decoys based on (**a**) the 43 test cases in the protein–RNA docking benchmark by Perez-Cano *et al.* (48) and (**b**) the 50 test cases in the protein–RNA docking benchmark by Huang and Zou (49).

Figure 7 shows the success rates of ITScore-PR for the two decoy sets. For comparison, the figure also shows the results of four other scoring methods that were computed by the Xiao group (62): RPDOCK, DARS-RNP (45), the potentials derived by Li *et al.* in the Wang Group (46) and DECK-RP (62,69). The figure displays similar trend that is observed in the aforementioned tests. ITScore-PR showed the best overall performance, especially when only the top few predictions were considered, which is often the case in realistic applications (Figure 7a and b). Specifically, for the docking decoys based on the benchmark of Perez-Cano *et al.* (48), ITScore-PR achieved a success rate of 25.6% (46.5%), compared with 22.7% (45.5%) for DECK-RP, 13.6% (36.4%) for DARS-RNP, 15.9% (27.3%) for the Li potential and 4.6% (27.3%) for RPDOCK if the top one prediction was considered (numerals in brackets indicate success rate for top 10 predictions; Supplementary Figure S7a). For the docking decoys based on the benchmark by Huang and Zou (49), ITScore-PR achieved a success rate of 48% (62%) versus 32% (52%) for DECK-RP, 38% (54%) for DARS-RNP, 10% (32%) for the Li potential and 26% (40%) for RPDOCK if the top one prediction was considered (numerals in brackets indicate success rate for top 10 predictions; Supplementary Figure S7b).

## CONCLUSION

In summary, we have developed an efficient scoring function for protein–RNA interactions based on a training set of diverse protein–RNA complex structures using a statistical mechanics-based iterative method, referred to as ITScore-PR. Our scoring function has been extensively assessed on its ability of identifying near-native structures on four different types of diverse test sets, compared with 10 other scoring methods. Overall, ITScore-PR showed a success rate as high as 86.1% (94.4%) for bound docking and 24% (46%) for (truly) unbound docking if the top one (10) prediction(s) was (were) considered. The fact that ITScore-PR performs much better for bound docking than for unbound docking indicates the necessity to consider molecular flexibility to further improve the discriminative power of ITScore-PR on selecting near-native structures. The scoring function can be used stand-alone or combined with other molecular docking software for protein–RNA recognition.

## AVAILABILITY

The bound and unbound docking decoy sets that were generated and optimized in this study using ITScore-PR for the 72 complexes in our protein–RNA docking benchmark are free to download at http://zoulab.dalton.missouri.edu/RNAdecoys.

## SUPPLEMENTARY DATA

Supplementary Data are available at NAR Online.

## FUNDING

National Institute of Health [R21GM088517]; National Science Foundation CAREER Award [DBI0953839]. Funding for open access charge: National Science Foundation CAREER Award [DBI0953839].

*Conflict of interest statement*. None declared.

## Supplementary Material

Supplementary Data
