# Supplementary material for: A knowledge-based scoring function for protein-RNA interactions derived from a statistical mechanics-based iterative method
Source: Nucleic Acids Res. 2014 Jan 28;42(7):e55. doi: 10.1093/nar/gku077 (PMC3985650; doi:10.1093/nar/gku077)
Supplement: Supplementary Data [file supp_42_7_e55__index.html]

A knowledge-based scoring function for protein-RNA interactions derived from a statistical mechanics-based iterative method — A knowledge-based scoring function for protein-RNA interactions derived from a statistical mechanics-based iterative method — Supplementary Data 

# A knowledge-based scoring function for protein-RNA interactions derived from a statistical mechanics-based iterative method

## Supplementary Data

files

**Files in this Data Supplement:**

- Supplementary Data - pdf file
