# Supplementary material for: A knowledge-based scoring function for protein-RNA interactions derived from a statistical mechanics-based iterative method
Source: Nucleic Acids Res. 2014 Jan 28;42(7):e55. doi: 10.1093/nar/gku077 (PMC3985650; doi:10.1093/nar/gku077)

Supplementary Data:

A knowledge-based scoring function for protein-RNA interactions derived from a statistical mechanics-based iterative method

Sheng-You Huang, and Xiaoqin Zou*

Department of Physics and Astronomy, Department of Biochemistry, Dalton Cardiovascular Research Center, and Informatics Institute, University of Missouri, Columbia, MO 65211

Key Words: Scoring function, protein-RNA interactions, molecular docking, statistical potential, molecular recognition.

Running title: An iterative knowledge-based scoring function for protein-RNA interactions

*Corresponding Author; Email: zoux@missouri.edu

Table S1: PDB codes of the 110 protein-RNA complexes in the training set for deriving ITScore-PR.

1A9N	1ASY	1B23	1B7F	1DI2	1DRZ	1EC6	1FEU	1I6U	1L9A	1M8W	1MFQ
1MFQ	1MJI	1MZP	1P6V	1RLG	1RPU	1SDS	1TFW	1TTT	1U6B	1VFG	1YYW
1YZ9	1ZBH	2AB4	2ASB	2BGG	2BS0	2BU1	2BX2	2D6F	2DER	2DLC	2E9T
2EZ6	2F8K	2GJE	2I82	2I91	2IZN	2NUE	2NZ4	2OZB	2PJP	2PY9	2W2H
2XLK	2Y8Y	2ZH6	2ZI0	2ZZN	3ADI	3AEV	3AGV	3AKZ	3AM1	3AMT	3BOY
3BSO	3BT7	3CUL	3CZ3	3DH3	3EGZ	3EQT	3HAX	3HHN	3I5X	3IAB	3IEV
3IVK	3K0J	3K49	3KFU	3KS8	3KTW	3L25	3MXH	3NCU	3NNH	3O7V	3OG8
3OIN	3QJJ	3QJL	3QSY	3R2C	3RW6	3SIU	3TRZ	3TS2	3TUP	3UMY	3V74
3V7E	3VJR	4DB2	4ED5	4ENN	4ERD	4FVU	4FY2	4GHA	4GHL	4H5P	5MSF
6MSF	7MSF										

Table S2: PDB codes of the test cases in the two RPDOCK docking decoy sets that are based on the protein-RNA docking benchmark by Perez-Cano et al. and the protein-RNA docking benchmark by Huang and Zou, respectively.

Decoy set	PDB codes
Based on the benchmark by Perez-Cano et al.	1DFU 2FMT 1U0B 1B7F 1E7K 1EC6 1HC8 1HVU 1JBR 1K8W 1KOG 1KQ2 1M8V 1M8W 1N78 1Q2R 1T4L 1U63 1WNE 1WPU 1WSU 1YVP 2AD9 2ADB 2ADC 2ASB 2AZ0 2BGG 2BH2 2BU1 2CZJ 2ERR 2F8K 2GJE 2GJW 2HGH 2I91 2QUX 2R7R 3BO2 3BSB 3BSX 3BX2
Based on the benchmark by Huang and Zou	1C0A 1DFU 1E8O 1F7Y 1FFY 1G1X 1GAX 1HQ1 1JBS 1JID 1K8W 1KOG 1LNG 1MMS 1N78 1Q2R 1QTQ 1R3E 1S03 1SJ3 1T0K 1YVP 2ANR 2AZ0 2BH2 2CSX 2CZJ 2FK6 2GJW 2QUX 2RFK 2XDB 2ZM5 2ZUE 3EPH 3FOZ 3HHZ 3LRR 3LWR 3MOJ 3OL9 3OVB 1R9F 1RC7 1UN6 2FMT 2VPL 2ZZM 3ADD 3FTF

Table S3: Protein-RNA atom pair occurrences in the native structures of the training database for deriving ITScore-PR. The rows refer to protein atom types. The columns stand for RNA atom types.

Protein	RNA atom types											
atom types	C2X	Car	C3X	N2N	N2X	Nar	N21	O2	O31	O32	O2-	P
C2+	2210	7498	10946	2235	1637	3154	1005	2161	2239	6503	4435	2219
C2-	1663	5713	9353	1689	1265	2488	815	1654	2067	5511	3791	1892
C2M	16957	54890	90928	17187	11421	23190	7406	17208	19801	53707	35816	17880
C2S	1956	5937	8746	1948	1256	2441	768	1925	1846	5233	3387	1713
Car	10539	33528	54646	10674	7485	14260	4415	10621	12434	31638	20960	10266
C3C	31019	100306	163501	31453	21594	42768	13574	31774	36189	96476	64376	31969
C3A	17259	56032	91925	17609	11749	23682	7546	17505	20035	54289	36064	17918
C3X	7933	26468	41418	8045	5679	11228	3610	7899	8816	24540	16462	8183
N2N	17262	55671	92447	17522	11538	23558	7513	17370	20012	54455	36141	18094
N2+	4547	15451	22130	4577	3372	6485	2054	4499	4563	13151	8916	4462
N2X	1921	5939	8702	1946	1265	2470	775	1910	1809	5142	3426	1723
Nar	1318	4268	6810	1344	982	1834	572	1342	1465	3992	2620	1316
N21	2083	7027	10464	2093	1513	2936	982	2019	2146	6270	4275	2147
N3+	2255	7710	11725	2288	1663	3289	1073	2244	2376	6970	4876	2384
O2M	16844	54203	89180	17040	11319	23112	7395	17092	19577	52972	35387	17612
O2S	1872	5838	8614	1906	1219	2393	767	1846	1785	5108	3351	1706
O3H	3139	10333	15848	3163	2194	4398	1376	3114	3436	9282	5989	3016
O2-	3376	11745	19088	3465	2591	5116	1669	3409	4226	11284	7758	3850
S31	160	585	875	169	128	279	97	167	212	501	306	155
S30	257	796	1403	258	189	354	111	275	300	840	568	285

Table S4: Score-RMSD correlation coefficients of ITScore-PR and five other scoring functions for different RMSD cutoffs, using the ROSETTA docking decoys generated by the Varani group. The results for DARS-RNP, QUASI-RNP, the Varani potential, and the Fernandez potential were taken from the study by Tuszynska and Bujnicki (ref. 45).

PDB code	RMSD (Å)	Correlation coefficient					
		**ITScore-PR**	dRNA	DARS-RNP	QUASI-RNP	Varani	Fernandez
1URN	5	0.84	0.74	0.77	0.7	0.37	-0.56
	10	0.89	0.82	0.83	0.79	0.27	-0.24
	20	0.84	0.81	0.81	0.79	0.21	-0.1
1E6C	5	0.79	0.75	0.81	0.79	0.57	-0.08
	10	0.86	0.87	0.9	0.89	0.38	-0.05
	20	0.80	0.84	0.87	0.86	0.31	-0.02
1FXL	5	0.97	0.96	0.94	0.95	0.87	0.61
	10	0.96	0.97	0.96	0.96	0.82	0.74
	20	0.92	0.94	0.93	0.94	0.7	0.83
1CVJ	5	0.99	0.96	0.96	0.96	0.5	0.85
	10	0.96	0.98	0.97	0.97	0.61	0.92
	20	0.92	0.94	0.93	0.93	0.46	0.9
1JID	5	0.70	0.63	0.58	0.58	0.35	0.33
	10	0.75	0.77	0.72	0.71	0.3	0.39
	20	0.71	0.76	0.7	0.69	0.27	0.29
Average	5	0.86	0.81	0.81	0.80	0.53	0.23
	10	0.89	0.88	0.88	0.87	0.47	0.35
	20	0.84	0.86	0.85	0.84	0.39	0.38

Table S5: The number of hits ($N_{\rm hits}$) and the best interface RMSD ($I_{\rm rmsd}$) of the hits in the bound and unbound RNA binding decoys for the 72 complexes in the protein-RNA docking benchmark by Huang and Zou.

PDB code	Type	Bound		Unbound		PDB code	Type	Bound		Unbound	
		$N_{\rm hits}$	$I_{\rm rmsd}$	$N_{\rm hits}$	$I_{\rm rmsd}$			$N_{\rm hits}$	$I_{\rm rmsd}$	$N_{\rm hits}$	$I_{\rm rmsd}$
1QTQ	easy	5	0.066	9	0.401	2XDB	easy	11	0.093	13	0.380
1FFY	easy	5	0.045	8	0.828	2ZNI	easy	7	0.059	5	1.556
1G1X	easy	8	0.150	7	1.075	2ZUE	easy	5	0.043	7	0.199
1N78	easy	7	0.084	7	1.945	1F7Y	easy	15	0.205	22	0.615
1GAX	easy	6	0.045	8	0.437	3CIY	easy	4	0.164	3	1.731
1H4S	easy	6	0.041	4	2.310	3DD2	easy	10	0.134	8	0.998
1SJ3	easy	2	0.079	10	0.380	3EPH	easy	17	0.080	16	0.251
1MMS	easy	27	0.054	19	1.685	3HHZ	easy	9	0.043	13	0.943
1S03	easy	14	0.203	18	1.243	1HQ1	easy	14	0.073	9	0.411
1J1U	easy	5	0.105	2	1.202	3LRR	easy	32	0.088	52	0.439
1JBS	easy	8	0.156	21	0.981	3MOJ	easy	31	0.195	32	0.978
1JID	easy	9	0.105	10	2.931	3OL9	easy	31	0.047	34	0.391
1K8W	easy	28	0.062	18	1.395	1E8O	easy	2	0.079	5	1.439
1KOG	easy	6	0.115	4	1.893	1F7U	medium	9	0.075	5	2.622
1LNG	easy	8	0.098	11	0.693	1IL2	medium	6	0.107	2	2.786
2CZJ	easy	8	0.357	4	3.023	1R9F	medium	21	0.166	3	2.900
1Q2R	easy	24	0.154	17	1.079	1SER	medium	4	0.182	5	2.732
2ZM5	easy	14	0.048	14	0.340	1UN6	medium	10	0.156	2	6.751
1R3E	easy	11	0.046	21	0.629	2UWM	medium	3	0.070	4	3.636
3LWR	easy	8	0.041	8	0.428	2ZZM	medium	11	0.113	11	1.607
1T0K	easy	15	0.242	22	1.113	2NUG	medium	12	0.024	14	3.096
3OVB	easy	17	0.087	26	0.385	2BTE	medium	5	0.185	4	3.155
3FOZ	easy	12	0.055	17	0.356	2ZKO	medium	22	0.157	1	5.157
1YVP	easy	17	0.103	9	1.763	2FMT	medium	3	0.095	2	2.501
1C0A	easy	4	0.060	4	1.012	2VPL	medium	23	0.125	9	2.062
2AKE	easy	5	0.177	3	0.737	1RC7	medium	20	0.197	25	4.406
2ANR	easy	5	0.107	9	0.327	3ADD	medium	6	0.074	7	2.557
2AZ0	easy	15	0.108	18	1.146	3FTF	medium	23	0.147	27	3.100
2BH2	easy	18	0.041	16	1.398	3HL2	medium	4	0.383	10	2.623
2CSX	easy	5	0.225	7	0.473	1H3E	difficult	3	0.136	2	21.279
2DU3	easy	2	0.174	3	0.612	2HW8	difficult	27	0.109	0	–
2FK6	easy	7	0.190	5	1.129	1OOA	difficult	12	0.125	2	6.138
1DFU	easy	32	0.127	59	0.948	1U0B	difficult	6	0.062	3	4.456
2GJW	easy	16	0.079	4	3.146	2IPY	difficult	3	0.062	0	–
2QUX	easy	7	0.044	2	0.654	2R8S	difficult	5	0.111	0	–
2RFK	easy	7	0.075	15	1.295	2V3C	difficult	7	0.100	0	–

Table S6: The rankings and accuracy qualities of the first successful predictions for the *bound* cases on the protein-RNA docking benchmark by Huang and Zou, in which the quality '1' stands for high accuracy, '2' for medium accuracy, '3' for acceptable accuracy and '4' for incorrect prediction based on the CAPRI criteria.

PDB	ITScore-PR		dRNA		DARS-RNP		QUASI-RNP		ZDOCK 2.1		PMF	
code	rank	quality	rank	quality	rank	quality	rank	quality	rank	quality	rank	quality
1QTQ	1	1	1	1	1	1	1	1	1	1	1	1
1FFY	1	1	1	1	1	1	1	1	1	1	1	1
1G1X	1	1	1	1	2	1	4	1	8	1	3	1
1N78	1	1	1	1	1	1	1	1	1	1	21	3
1GAX	1	1	1	1	1	1	1	1	1	1	30	1
1H4S	1	1	11	1	10	1	16	1	8	2	31	3
1SJ3	1	1	1	1	2	1	5	1	7	2	1	1
1MMS	1	1	1	1	1	1	1	1	1	1	25	3
1S03	1	1	1	1	2	1	4	1	2	1	19	3
1J1U	1	1	201	1	17	1	254	1	131	2	265	2
1JBS	11	1	3	3	1	2	1	1	57	3	253	3
1JID	1	1	1	1	5	1	13	1	67	2	11	3
1K8W	1	1	1	1	1	1	1	1	1	1	55	3
1KOG	1	1	26	1	3	1	40	1	2	1	14	3
1LNG	1	1	1	1	1	1	1	1	2	1	6	1
2CZJ	1	1	14	1	1	1	6	1	2	1	132	3
1Q2R	1	1	1	1	1	1	1	1	1	1	21	3
2ZM5	1	1	1	1	1	1	2	1	1	1	7	1
1R3E	1	1	1	1	1	1	1	1	1	1	1381	1
3LWR	1	1	1	1	1	1	1	1	1	1	120	3
1T0K	4	1	18	3	4	1	11	3	121	3	326	3
3OVB	1	1	1	1	3	1	7	1	1	1	29	1
3FOZ	1	1	1	1	1	1	2	1	1	1	1	1
1YVP	1	1	7	1	1	1	1	1	1	1	501	3
1C0A	1	1	1	1	1	1	1	1	2	1	1	1
2AKE	9	1	339	2	83	2	179	2	9	2	—	4
2ANR	2	1	53	1	63	1	131	1	81	3	1324	3
2AZ0	1	1	1	1	1	1	1	1	1	2	2	2
2BH2	1	1	1	1	1	1	1	1	1	1	1	1
2CSX	1	1	1	1	1	1	1	1	22	3	1	1
2DU3	12	1	565	1	200	1	313	1	178	2	—	4
2FK6	6	3	106	3	67	3	52	3	79	2	4	3
1DFU	1	1	1	1	1	1	1	1	1	2	1	1
2GJW	1	1	1	1	1	1	1	1	1	1	2	1
2QUX	1	1	1	1	4	1	5	1	63	1	41	3
2RFK	1	1	1	1	1	1	1	1	6	1	8	3
2XDB	2	1	5	1	2	1	4	1	77	2	487	1
2ZNI	1	1	1	1	1	1	1	1	1	1	1	1
2ZUE	1	1	1	1	1	1	2	1	1	1	2	1

Continued on Next Page…

Table S6 – Continued

PDB	ITScore-PR		dRNA		DARS-RNP		QUASI-RNP		ZDOCK 2.1		PMF	
code	rank	quality	rank	quality	rank	quality	rank	quality	rank	quality	rank	quality
1F7Y	1	1	1	1	1	1	1	1	1	1	1	1
3CIY	1	1	14	1	1	1	2	1	21	2	603	2
3DD2	1	1	1	1	2	1	1	1	5	1	34	1
3EPH	1	1	1	1	1	1	1	1	1	1	1	1
3HHZ	1	1	13	1	51	3	33	3	674	3	2	2
1HQ1	1	1	1	1	1	1	2	1	2	1	508	3
3LRR	2	1	34	3	93	3	36	3	14	2	2	3
3MOJ	1	1	9	3	34	3	28	3	24	3	433	3
3OL9	1	1	1	1	3	1	9	1	1	1	1332	1
1E8O	1	1	2	1	68	1	30	1	27	3	1	1
1F7U	1	1	1	1	1	1	1	1	1	1	1	1
1IL2	1	1	1	1	1	1	2	1	1	1	3	1
1R9F	1	1	1	1	25	1	66	1	15	1	3	2
1SER	1	1	1	1	9	1	23	1	116	3	8	1
1UN6	1	1	2	3	9	1	28	1	11	3	1	1
2UWM	18	1	552	1	52	1	101	1	143	2	128	1
2ZZM	1	1	1	1	1	1	1	1	1	1	1715	1
2NUG	1	1	1	1	2	1	2	1	1	3	1	1
2BTE	1	1	1	1	3	1	20	1	315	1	12	1
2ZKO	1	1	1	1	2	1	3	1	2	1	2	3
2FMT	1	1	1	1	2	1	5	1	957	3	20	3
2VPL	1	1	1	1	1	1	2	1	1	1	5	2
1RC7	1	1	1	1	1	1	11	1	3	3	2	3
3ADD	1	1	1	1	16	1	36	1	256	1	1	1
3FTF	1	1	1	1	1	1	4	1	41	2	4	3
3HL2	63	3	187	3	26	3	36	3	1085	3	59	3
1H3E	1	1	2	1	8	1	11	1	7	2	728	1
2HW8	1	1	1	1	1	1	3	1	1	1	1	1
1OOA	1	1	20	1	28	1	72	1	6	2	68	3
1U0B	1	1	1	1	1	1	1	1	1	1	44	1
2IPY	1	1	1	1	1	1	1	1	6	1	1472	1
2R8S	1	1	1	1	3	1	5	1	7	1	23	1
2V3C	1	1	1	1	2	1	2	1	73	2	541	2

Table S7: The rankings and accuracy qualities of the first successful predictions for the non-native complex test cases on the protein-RNA docking benchmark by Huang and Zou.

PDB	ITScore-PR		dRNA		DARS-RNP		QUASI-RNP		ZDOCK 2.1		PMF	
code	rank	quality	rank	quality	rank	quality	rank	quality	rank	quality	rank	quality
1QTQ	1	1	1	1	1	1	1	1	1	1	2	1
1FFY	1	1	1	1	1	1	2	1	1	2	1	1
1G1X	1	2	2	2	4	2	13	2	10	2	3	2
1N78	1	2	1	2	1	2	1	2	1	2	47	3
1GAX	1	1	1	1	1	1	1	1	1	1	18	1
1H4S	89	2	213	3	118	2	278	2	302	3	98	3
1SJ3	1	1	3	1	2	1	27	3	2	2	6	1
1MMS	1	2	1	2	1	2	1	2	3	2	70	3
1S03	1	2	1	2	8	2	12	2	5	2	119	3
1J1U	858	2	1245	2	621	2	1219	2	823	2	—	4
1JBS	9	3	67	2	1	2	5	2	18	3	713	3
1JID	60	3	71	2	116	3	24	3	40	3	82	3
1K8W	2	2	4	2	16	2	28	2	4	2	152	3
1KOG	116	3	417	3	167	2	636	2	339	2	29	3
1LNG	1	1	1	1	1	1	1	1	1	2	71	3
2CZJ	392	3	463	3	47	3	225	3	229	2	—	4
1Q2R	4	3	1	2	1	2	1	2	1	2	1646	2
2ZM5	1	1	1	1	1	1	1	1	1	1	3	1
1R3E	1	1	1	1	1	1	1	1	1	1	26	3
3LWR	1	1	1	1	1	1	1	1	1	1	37	2
1T0K	127	3	46	3	59	3	115	3	43	3	—	4
3OVB	1	1	1	1	2	1	7	2	1	1	7	1
3FOZ	1	1	1	1	1	1	1	1	1	1	5	1
1YVP	32	3	435	2	17	2	45	2	60	2	346	3
1C0A	1	2	5	2	1	2	1	2	55	2	1	2
2AKE	41	2	480	2	244	2	485	2	574	2	—	4
2ANR	35	1	151	1	199	1	222	3	307	3	—	4
2AZ0	2	3	2	1	3	1	1	1	9	2	1	3
2BH2	1	2	1	2	1	2	3	2	1	2	87	2
2CSX	1	1	1	2	1	2	1	2	20	2	1	1
2DU3	134	1	1165	1	509	1	700	1	557	3	—	4
2FK6	104	2	266	2	752	2	600	3	470	3	31	3
1DFU	2	2	1	2	4	2	3	2	1	3	3	2
2GJW	211	3	335	3	112	3	328	3	113	3	—	4
2QUX	1	1	5	1	28	1	34	1	178	3	61	3
2RFK	1	2	1	2	1	2	4	2	2	2	2	3
2XDB	3	1	18	1	10	3	32	3	24	3	258	1
2ZNI	123	2	6	2	2	2	8	2	69	3	1826	2
2ZUE	1	1	1	1	1	1	1	1	1	2	1	1
1F7Y	1	1	1	1	2	1	3	1	1	2	75	3
3CIY	381	2	698	2	252	2	272	2	—	4	873	3

Continued on Next Page…

Table S7 – Continued

PDB	ITScore-PR		dRNA		DARS-RNP		QUASI-RNP		ZDOCK 2.1		PMF	
code	rank	quality	rank	quality	rank	quality	rank	quality	rank	quality	rank	quality
3DD2	13	3	6	3	24	1	15	3	52	2	26	3
3EPH	1	1	1	1	1	1	1	1	1	1	1	1
3HHZ	4	3	36	3	13	3	4	3	66	3	11	2
1HQ1	1	1	1	1	4	1	25	1	20	1	200	3
3LRR	2	1	21	3	60	3	73	3	8	3	3	3
3MOJ	71	3	75	3	85	3	84	3	86	3	489	3
3OL9	1	1	1	1	3	1	6	1	1	1	1458	1
1E8O	54	2	59	2	671	2	215	2	431	3	4	2
1F7U	1249	2	1	2	1	2	1	2	2	2	—	4
1IL2	1674	3	43	3	7	3	26	3	—	4	—	4
1R9F	84	3	23	3	352	2	370	3	73	2	222	3
1SER	18	2	12	2	344	2	287	3	97	3	1	3
1UN6	1175	3	113	3	123	3	140	3	754	3	1	3
2UWM	443	3	216	3	497	3	189	3	334	3	348	3
2ZZM	1	2	1	2	1	2	1	2	3	3	1763	2
2NUG	2	2	2	2	2	2	2	2	2	3	1340	2
2BTE	3	2	1	3	30	3	44	3	21	3	231	3
2ZKO	383	3	—	4	—	4	—	4	810	3	—	4
2FMT	161	2	91	2	140	2	158	2	—	4	70	3
2VPL	6	2	11	2	77	3	132	3	305	2	505	3
1RC7	2	2	3	3	29	2	75	3	35	2	9	3
3ADD	10	2	4	2	65	2	116	2	27	3	17	3
3FTF	1	2	1	2	8	2	9	2	91	2	24	3
3HL2	433	3	609	2	12	3	2	3	388	3	82	3
1H3E	6	2	351	2	120	2	251	2	917	2	57	3
2HW8	—	4	—	4	—	4	—	4	—	4	—	4
1OOA	780	3	129	3	977	3	1026	3	314	3	—	4
1U0B	194	3	15	3	89	3	165	3	—	4	—	4
2IPY	—	4	—	4	—	4	—	4	—	4	—	4
2R8S	—	4	—	4	—	4	—	4	—	4	—	4
2V3C	—	4	—	4	—	4	—	4	—	4	—	4

Table S8: PDB codes of the 50 'truly' unbound test cases from the protein-RNA docking benchmark by Huang and Zou in which the unbound protein (RNA) is in either free form or bound to an RNA (a protein) that has a sequence identity of $< 70\%$ ($< 30\%$) with the RNA (protein) in the corresponding native complex.

1C0A	1DFU	1F7Y	1G1X	1H4S	1J1U	1JBS	1JID	1K8W	1KOG	1N78	1Q2R
1R3E	1S03	1SJ3	1T0K	1YVP	2AZ0	2BH2	2CZJ	2FK6	2GJW	2QUX	2ZNI
3CIY	3DD2	3LRR	3MOJ	3OL9	1F7U	1IL2	1R9F	1RC7	1SER	2BTE	2FMT
2NUG	2UWM	2VPL	2ZKO	2ZZM	3FTF	3HL2	1H3E	1OOA	1U0B	2HW8	2IPY
2R8S	2V3C										

Table S9: The rankings and accuracy qualities of the first successful predictions for the *unbound/model* cases on the protein-RNA docking benchmark by Perez-Cano et al.

PDB	ITScore-PR		dRNA		DARS-RNP		QUASI-RNP		ZDOCK 2.1		PMF	
code	rank	quality	rank	quality	rank	quality	rank	quality	rank	quality	rank	quality
1C0A	136	3	61	3	37	3	22	3	325	3	—	4
1C9S	—	4	—	4	—	4	—	4	—	4	—	4
1DFU	1	3	3	3	9	3	14	3	9	3	151	3
1DK1	6	3	1	2	62	3	110	3	48	3	14	3
1E7K	53	2	45	2	42	3	44	3	75	3	43	3
1E8O	1336	3	1300	3	1386	3	1401	3	372	3	215	3
1EFW	42	2	114	2	149	2	261	2	673	2	385	3
1EKZ	507	3	679	3	416	3	275	3	38	3	771	3
1F7U	144	3	1	2	1	2	1	2	151	2	297	3
1G1X	16	3	316	3	13	3	16	3	33	2	60	3
1H3E	8	2	450	2	268	2	425	2	1249	2	—	4
1H4S	607	3	198	3	556	3	562	3	298	3	46	3
1HC8	1	3	8	3	20	3	84	3	170	3	95	3
1HQ1	86	3	26	3	209	3	162	3	395	3	—	4
1HVU	10	3	9	3	61	3	174	3	79	3	13	3
1J1U	865	2	911	2	841	2	969	2	—	4	—	4
1J2B	—	4	—	4	—	4	—	4	—	4	—	4
1JBR	1	3	42	3	6	3	3	3	5	2	501	3
1K1G	28	3	14	3	15	3	34	3	28	3	—	4
1K8W	1	2	4	2	1	2	1	2	8	2	65	3
1KOG	499	3	484	3	669	3	554	3	523	3	215	3
1KQ2	3	2	4	2	3	2	3	2	3	2	1	2
1LNG	192	3	33	3	123	3	95	3	675	3	—	4
1M5O	3	2	647	2	12	2	74	2	1254	3	135	3
1M8V	178	3	268	3	272	3	308	3	298	3	2	3
1MMS	101	3	39	3	47	3	158	3	299	3	—	4
1N78	1	2	1	2	1	2	1	2	4	2	17	2
1OOA	96	3	2	3	18	3	11	3	3	3	—	4
1Q2R	3	3	2	2	3	2	1	2	2	2	43	3
1QF6	1747	2	7	2	1	2	1	2	—	4	1841	3
1QTQ	—	4	—	4	—	4	—	4	1727	3	—	4
1R3E	1	3	5	3	26	3	62	3	138	3	7	3
1RKJ	—	4	—	4	—	4	—	4	309	3	—	4
1S03	1	3	1	3	19	3	31	3	23	3	24	3
1SER	30	2	29	2	384	2	407	2	77	3	2	3
1T0K	916	3	1236	3	805	3	1083	3	687	3	—	4
1T4L	5	3	1	3	3	3	23	3	12	3	6	3
1U0B	296	3	94	3	724	3	796	3	—	4	—	4
1U63	1	2	5	2	27	2	62	2	44	2	7	2
1WNE	1	1	1	1	1	2	2	3	1	1	1056	3
1WPU	4	1	6	1	11	1	3	1	1	1	—	4

Continued on Next Page…

Table S9 – Continued

PDB	ITScore-PR		dRNA		DARS-RNP		QUASI-RNP		ZDOCK 2.1		PMF	
code	rank	quality	rank	quality	rank	quality	rank	quality	rank	quality	rank	quality
1WSU	227	3	62	3	253	3	128	3	90	3	19	2
1YVP	178	3	537	3	63	3	130	3	495	3	—	4
2AD9	4	2	3	3	1	3	1	3	4	3	4	2
2ADB	1	3	1	3	1	2	3	3	15	3	1	3
2ADC	3	3	3	3	4	3	11	3	25	3	1	3
2AZ0	1	3	3	1	7	1	1	1	1	2	1	3
2B3J	1070	3	28	3	16	3	11	3	382	3	—	4
2BH2	1	2	1	2	1	2	3	2	1	2	61	3
2BTE	105	3	31	3	139	3	126	3	268	3	—	4
2CJK	48	3	92	3	164	3	554	3	238	3	—	4
2CSX	68	2	21	2	102	2	249	2	240	2	46	3
2CZJ	373	2	440	2	99	2	288	2	101	2	798	3
2DB3	—	4	—	4	—	4	—	4	—	4	—	4
2DU3	430	3	1422	3	1366	3	1451	3	—	4	—	4
2ERR	17	2	3	3	1	1	4	1	35	3	129	3
2FMT	161	2	91	2	140	2	158	2	—	4	70	3
2FY1	1	2	7	3	12	3	16	3	28	3	—	4
2GJW	211	3	335	3	112	3	328	3	113	3	—	4
2HGH	—	4	—	4	—	4	—	4	—	4	—	4
2HW8	—	4	—	4	—	4	—	4	488	2	—	4
2IPY	—	4	—	4	—	4	—	4	—	4	—	4
2IX1	1	2	1	2	122	2	122	2	1	2	481	2
2JPP	965	3	145	3	51	3	103	3	208	3	—	4
2QUX	1	1	8	1	26	1	29	1	189	3	367	1
2R7R	3	3	4	3	8	3	19	3	4	3	436	3
2R8S	777	3	888	3	828	3	1022	3	366	2	—	4
2V3C	—	4	—	4	—	4	—	4	—	4	—	4
2ZKO	280	3	104	3	177	3	277	3	172	3	712	3
3BSB	1	3	1	2	1	2	1	2	1	2	1	3
3BX2	4	3	5	3	1	3	2	3	12	2	68	3
3CIY	381	2	698	2	252	2	272	2	—	4	873	3

Figure S1: The success rate (red circles) and the average RMSD (black squares) of the predicted binding modes as a function of the iterative step for the derivation of ITScore-PR. The dashed line stands for the success rate of 100%.

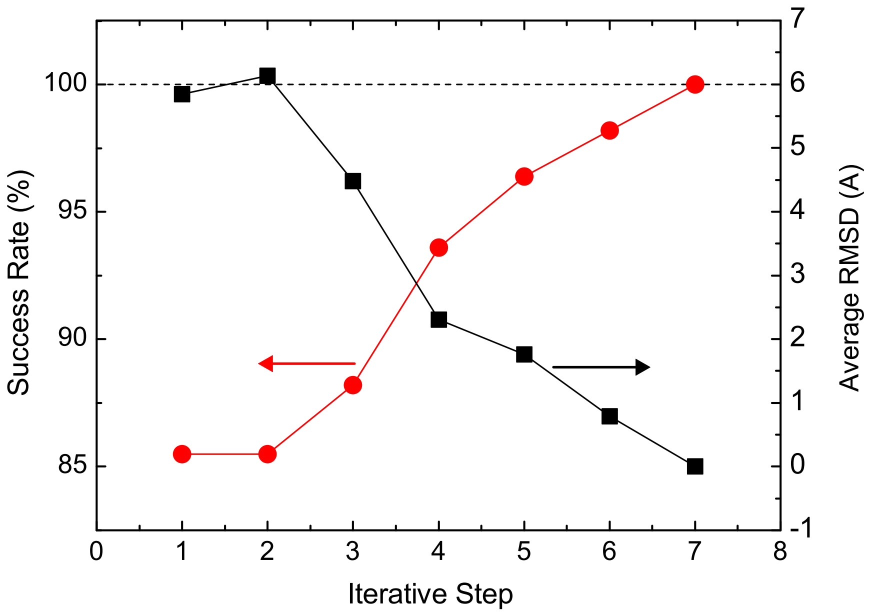

Figure S2: The RMSD distribution of the RNA binding decoys for the bound (A) and unbound (B) test case of target 1DFU from the protein-RNA docking benchmark by Huang and Zou.

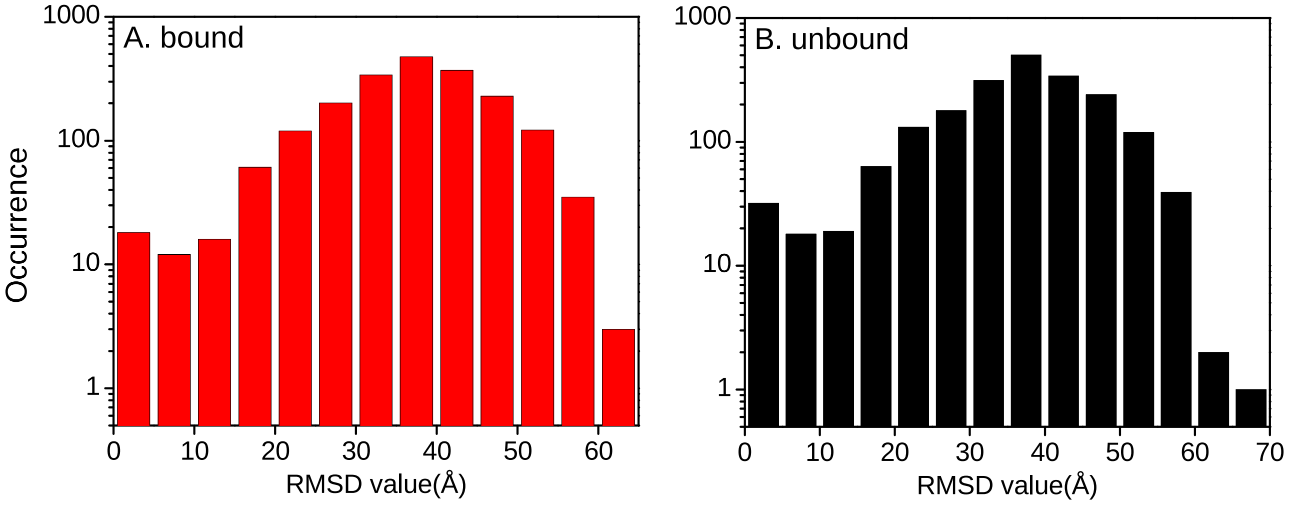

Figure S3: An illustration of sampled binding orientations of the RNA molecule (green) around the protein (magenta) for the bound (A) and unbound (B) case of target 1DFU from the protein-RNA docking benchmark by Huang and Zou. The binding positions of the RNA are roughly represented by the centers of the RNA binding modes, where the hits are highlighted in red.

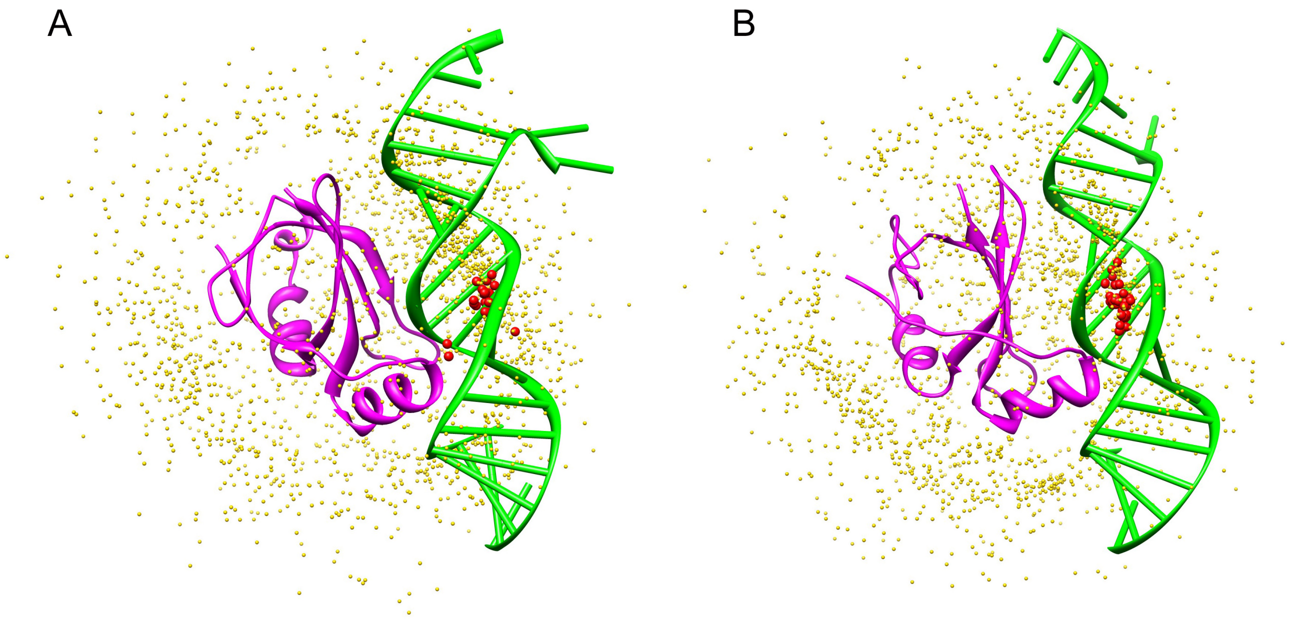

Figure S4: The success rates of ITScore-PR and five other scoring functions (dRNA, DARS-RNP, QUASI-RNP, ZDOCK 2.1 and PMF) for the bound test cases of the 72 complexes in the protein-RNA docking benchmark by Huang and Zou when the top one and top ten ranked predictions were considered, respectively.

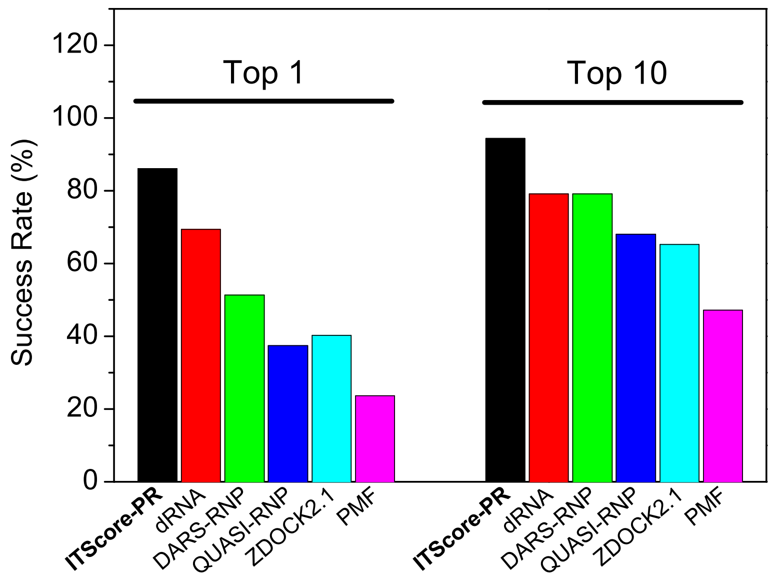

Figure S5: The success rates of ITScore-PR and five other scoring functions (dRNA, DARS-RNP, QUASI-RNP, ZDOCK 2.1 and PMF) for (a) the non-native test cases of the 72 complexes and (b) the 'truly' unbound test cases of the 50 complexes from the protein-RNA docking benchmark by Huang and Zou when the top one and top ten ranked predictions were considered, respectively.

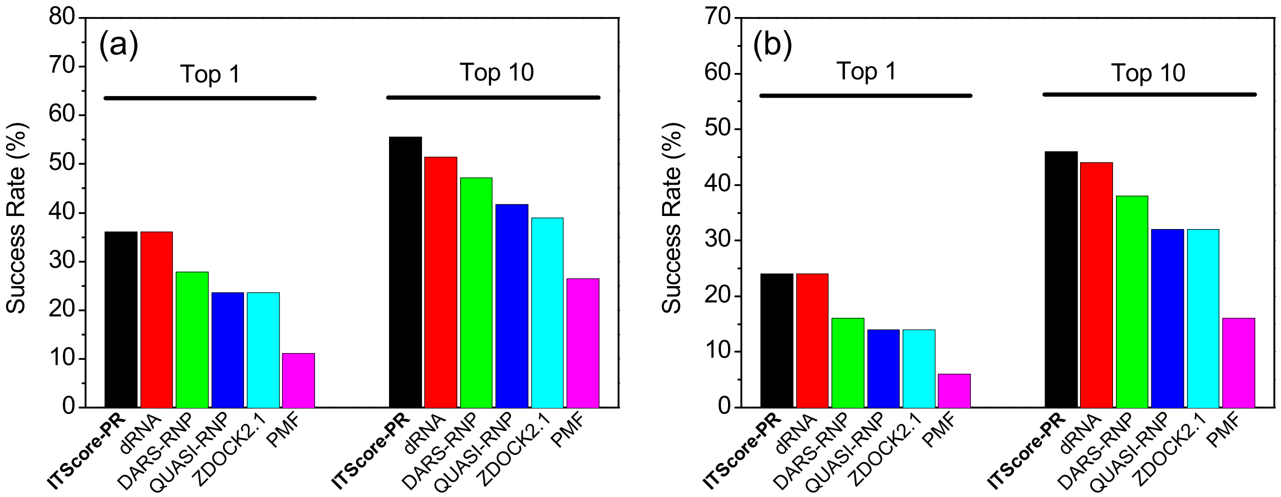

Figure S6: The success rates of ITScore-PR and five other scoring functions (dRNA, DARS-RNP, QUASI-RNP, ZDOCK 2.1 and PMF) for the unbound test cases of the 72 complexes from the protein-RNA docking benchmark by Perez-Cano et al. when the top one and top ten ranked predictions were considered, respectively.

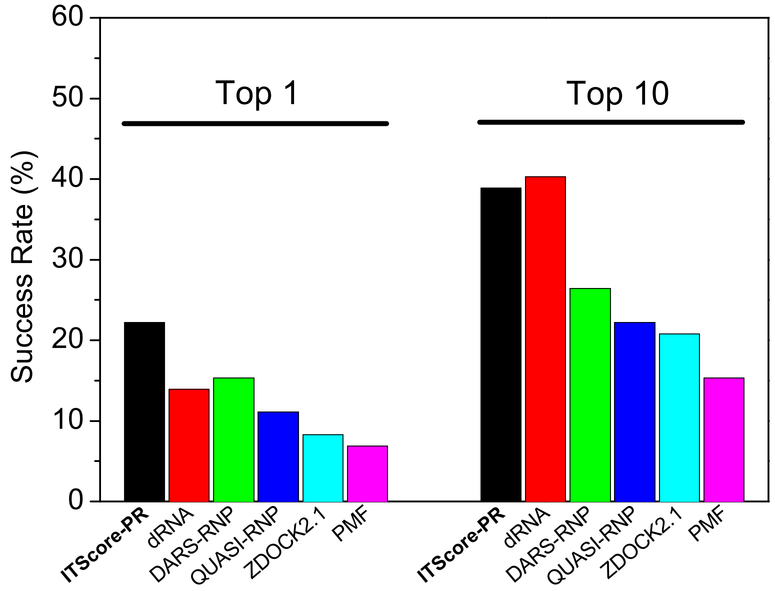

Figure S7: The success rates of ITScore-PR and four other scoring functions (DECK-RP, DARS-RNP, the Li potential and RPDOCK) for the RPDOCK docking decoys based on (a) the 43 test cases in the protein-RNA docking benchmark by Perez-Cano et al. and (b) the 50 test cases in the protein-RNA docking benchmark by Huang and Zou when the top one and top ten ranked predictions were considered, respectively.

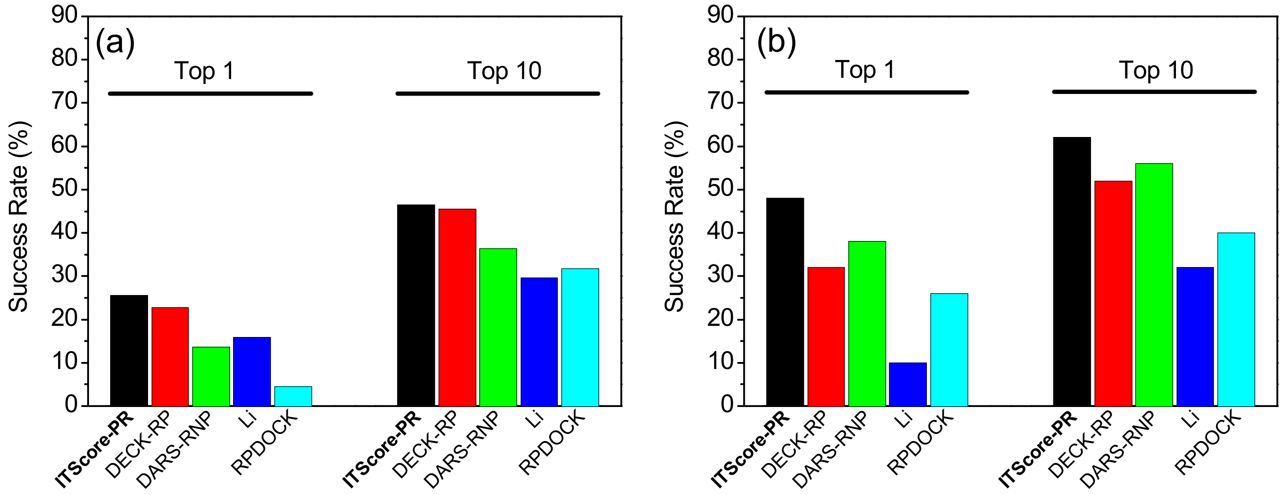

Supplement: Supplementary Data [file supp_gku077_nar-03122-met-g-2013-File002.pdf]
